# Transcriptome Analysis of the Breast Muscle of Xichuan Black-Bone Chickens Under Tyrosine Supplementation Revealed the Mechanism of Tyrosine-Induced Melanin Deposition

**DOI:** 10.3389/fgene.2019.00457

**Published:** 2019-05-15

**Authors:** Donghua Li, Xinlei Wang, Yawei Fu, Chenxi Zhang, Yanfang Cao, Jie Wang, Yanhua Zhang, Yuanfang Li, Yi Chen, Zhuanjian Li, Wenting Li, Ruirui Jiang, Guirong Sun, Yadong Tian, Guoxi Li, Xiangtao Kang

**Affiliations:** ^1^College of Animal Science and Veterinary Medicine, Henan Agricultural University, Zhengzhou, China; ^2^Henan Innovative Engineering Research Center of Poultry Germplasm Resource, Henan Agricultural University, Zhengzhou, China

**Keywords:** breast muscle, chicken, melanin, melanocytes, tyrosine, transcriptome

## Abstract

The Xichuan black-bone chicken, which is a rare local chicken species in China, is an important genetic resource of black-bone chickens. Tyrosine can affect melanin production, but the molecular mechanism underlying tyrosine-induced melanin deposition in Xichuan black-bone chickens is poorly understood. Here, the blackness degree and melanin content of the breast muscle of Xichuan black-bone chickens fed a basic diet with five levels of added tyrosine (i.e., 0.2, 0.4, 0.6, 0.8, and 1.0%; these groups were denoted test groups I-V, respectively) were assessed, and the results showed that 0.8% tyrosine was the optimal level of added tyrosine. Moreover, the effects of tyrosine supplementation on the proliferation and tyrosinase content of melanocytes in Xichuan black-bone chickens were evaluated. The results revealed a dose-dependent relationship between tyrosine supplementation and melanocyte proliferation. In addition, 417 differentially expressed genes (DEGs), including 160 upregulated genes and 257 downregulated genes, were identified in a comparative analysis of the transcriptome profiles constructed using the pooled total RNA from breast muscle tissues of the control group and test group IV, respectively (fold change ≥2.0, *P* < 0.05). These DEGs were mainly involved in melanogenesis, the calcium signaling pathway, the Wnt signaling pathway, the mTOR signaling pathway, and vascular smooth muscle contraction. The pathway analysis of the DEGs identified some key genes associated with pigmentation, such as *DCT* and *EDNRB2*. In summary, the melanin content of breast muscle could be markedly enhanced by adding an appropriate amount of tyrosine to the diet of Xichuan black-bone chickens, and the *EDNRB2*-mediated molecular regulatory network could play a key role in the biological process of tyrosine-induced melanin deposition. These results have deepened the understanding of the molecular regulatory mechanism of melanin deposition in black-bone chickens and provide a basis for the regulation of nutrition and genetic breeding associated with melanin deposition in Xichuan black-bone chickens.

## Introduction

The black-bone chicken is an important resource in poultry production, and the fact that its body contains melanin significantly differentiates it from other chicken breeds (Tian et al., 2007; Tu et al., 2009b; Yu et al., 2018a). Many studies have shown that black-bone chickens have medical benefits, such as antioxidant activity (Xin et al., 2009) and the abilities to delay aging (Xu et al., 1999), treat anemia (Xie et al., 2009) and cure female menstrual abnormalities (Tu et al., 2009a). Due to their unique edible and medicinal value, black-bone chickens are highly preferable by consumers, and the exploitation and genetic breeding of black-bone chicken resources have become increasingly important. It is known that the melanin content in the meat, skin and bone of black-bone chickens is closely related to its product value and medicinal effects (Yu et al., 2018b). Therefore, the improvement of melanin deposition by nutritional regulation and genetic breeding is of great significance for the exploitation and utilization of black-bone chicken resources; however, the relevant research remains scarce.

Melanin is a type of high-protein molecule synthesized from melanin bodies in melanocytes (Diffey et al., 1995; Mackintosh, 2001), and studies have shown that nutritional factors can affect the deposition of melanin in the body (Ralli and Graef, 1943; Li et al., 2011). The ingestion of nutrients as a signal induces transcription, RNA processing and stability, protein synthesis and modification, which are processes that affect DNA replication, regulate gene expression, and maintain cell proliferation, differentiation and adaptation (Walker and Blackburn, 2004; Ruemmele and Garnier-Lengliné, 2012). Therefore, the addition of exogenous nutrition can effectively regulate the deposition of melanin in animals. In black-bone chickens, melanin is produced by tyrosinase through a series of biochemical reactions (Sato et al., 2007). Tyrosine, a type of aromatic amino acid, is a precursor of melanin synthesis (Scheiner et al., 2002), and black-bone chickens might have a precise synergistic regulatory mechanism between tyrosine metabolism and melanin synthesis that regulates the synthesis and deposition of melanin. The elucidation of the above mechanism is of great significance for improving the meat quality of black-bone chickens through the regulation of nutrition and for identifying the molecular markers related to melanin deposition in the breeding of black-bone chickens. However, the molecular mechanism underlying tyrosine-induced melanin deposition in black-bone chickens remains poorly understood.

The Xichuan black-bone chicken, which is a rare local chicken species, is mainly distributed in Xichuan County, Henan Province, China. This variety has five black parts (beak, skin, bones, legs, and meat) and is an important genetic resource of black-bone chickens. Because its meat is tender and delicious and has high nutritional and medicinal value, this variety has become an important object of production and development in recent years. Increased production performance and the production of high-quality poultry meat with a high content of melanin through nutritional regulation or genetic improvement are currently the main goal of the resource exploitation of Xichuan black-bone chickens. The breast muscle is an important part of skeletal muscle and the main source of poultry meat. Therefore, in this study, the breast muscle of Xichuan black-bone chicken was used as the research material to evaluate the effects of different concentrations of tyrosine on melanin deposition and to analyze the transcriptome profiles of breast muscle under tyrosine supplementation. We also verified the effect of tyrosine on the proliferation and tyrosinase content of melanocytes and determined the key genes and molecular regulatory network associated with tyrosine-induced melanin deposition. The main objectives of this study were to optimize the optimal supplementary amount of tyrosine in feed for the promotion of melanin deposition in the breast muscle of Xichuan black-bone chickens and to clarify the potential molecular mechanism underlying tyrosine-induced melanin deposition in black-bone chickens. The results obtained in this study will provide a better understanding of the molecular regulatory mechanisms of melanin deposition in black-bone chickens and contribute to nutrient regulation and genetic breeding related to melanin deposition in Xichuan black-bone chickens.

## Materials and Methods

### Ethics Statement

All sample collections and treatments were conducted strictly in accordance with the protocol approved by the Institutional Animal Care and Use Committee (IACUC) of Henan Agricultural University, China (11-0099).

### Experimental Animals and Design

A total of 480 one-day-old healthy and weight-matched Xichuan black-bone chickens were randomly divided into six groups (the control group and test groups I-V), with five biological replicates of each group and 16 chickens per biological replicate. The control group was fed a basal diet (Supplementary Table S1). For test groups I-V, the basal diet was supplemented with 0.2, 0.4, 0.6, 0.8, and 1.0% tyrosine, respectively. Tyrosine (99%) was obtained from Swire Coca-Cola Drinks Co., Ltd., (Zhengzhou, Henan Province, China), and the trial period lasted 12 weeks. The chickens in all the treatment groups were fed and managed under the same conditions and were allowed free access to feed and water during the experimental period. The rooms were regularly cleaned and disinfected, and the chickens were routinely immunized.

### Sample Collection

At the end of the feeding trial, all the experimental chickens were fasted for 12 h with continued access to water. Two Xichuan black-bone chickens from each replicate (a total of 60 chickens) were randomly selected. The live weight and dressing percentage of the 60 chickens were measured. These experimental chickens were euthanized by intravenous injection of KCl (1–2 mg/kg) under deep anesthesia, and 1–2 g of breast meat was frozen immediately in liquid nitrogen and stored at -80°C until RNA-seq analysis. The remaining breast meat was stripped from both sides for analysis of the melanin content.

### Evaluation of Melanin Deposition in Breast Muscle

First, the breast muscle color of each experimental chicken was determined with a portable NR10QC colorimeter (3nh, China). The color parameter was measured based on the lightness index (L^∗^). All color values were obtained from three areas of each breast muscle. A higher reading indicated a lower degree of blackness, and vice versa. Second, the melanin content in the breast muscle of each experimental chicken was analyzed. Briefly, 50-g breast muscle samples from each chicken were weighed, and after the fascia, membrane and fat were removed, the samples were heated in an oven at 65°C for 24 h. The crushed samples were hydrolyzed with papain under neutral conditions for 3 h during heating at 55°C in a water bath, and after centrifugation, the precipitates were separated, soaked in 200 mL of 6 mol/L HCl and heated in an electric furnace for 30 min. The residue was collected, washed, wrapped with filter paper and placed in a Soxhlet extraction tube. Subsequently, the residue was degreased with ether in a water bath at 42°C, and the filter paper was washed repeatedly with distilled water and dried at 80°C in an oven. The melanin was removed and weighed using a microbalance. The melanin content (as the percentage of fresh tissue weight) was then calculated as follows: melanin weight (g)/sample weight (g) × 100%.

### Exposure of Chicken Melanocytes to Tyrosine and Evaluation of the Effects of Tyrosine Exposure

To evaluate the effect of tyrosine on melanin deposition at the cellular level, we exposed melanocytes from Xichuan black-bone chickens to different concentrations of tyrosine. The initial density of the cells was 1 × 10^4^ cells/mL. The control group was cultured with normal melanocyte medium, and the experimental groups were cultured with medium containing different concentrations of tyrosine (10^-9^, 10^-8^, 10^-7^, and 10^-6^). Fourth-generation melanocytes at the logarithmic phase of growth were digested with 0.25% trypsin (Gibco Company, United States) and seeded into cell culture plates for follow-up testing. On the one hand, the effect of tyrosine on the proliferation of melanocytes was evaluated by the Cell Counting Kit-8 (CCK-8) method on 96-well cell culture plates. Ten replicate wells were used for each group. Ten microliters of CCK-8 solution was added to each well, and the plates were incubated for 2 h at 37°C in an atmosphere with 5% CO_2_. The absorbance of each well at 450 nm was measured using a high-throughput multifunctional microplate test system. The proliferation rate was calculated as follows: [(A450 (with tyrosine) – A450 (medium only)]/[A450 (without tyrosine) – A450 (medium only)] × 100%. On the other hand, the effect of tyrosine on the tyrosinase content in melanocytes was detected using a chicken tyrosinase ELISA kit on six-well plates. Each group was tested in six replicate wells in triplicate. The plates were incubated for 72 h in a humidified 5% CO_2_ incubator at 37°C, and the intracellular components were then extracted using an ultrasonic cell disrupter. The content of tyrosinase in the cells was detected in strict accordance with the instructions provided with the tyrosinase ELISA kit. Moreover, the effects of tyrosine on endothelin receptor B subtype 2 (EDNRB2) gene expression in the above-treated melanocytes were also determined by qRT-PCR analysis.

### Transcriptome Sequencing and Annotation of Breast Muscle

Based on the results of the melanin determination, the highest amount of melanin deposition in breast muscle tissue was detected in test group IV (0.8% dietary tyrosine level). Therefore, breast muscle samples from the control group and test group IV were selected for transcriptome sequencing. The total RNA from the breast muscle samples was extracted using an RNA extraction kit (Takara, Dalian, China) following the appropriate procedure and were processed using DNase I (RNase Free, QIAGEN). To characterize a general overview of genes expressed in the breast muscle tissues of the highest amount of melanin deposition, the pooled total RNA from three individuals of test group IV and the control group was used for cDNA library construction, respectively. The libraries were generated using the NEBNext^®^ Ultra^TM^ Directional RNA Library Prep Kit for Illumina^®^ (NEB, Ipswich, MA, United States). The processes necessary for library construction, such as mRNA isolation, fragmentation, first-strand cDNA synthesis, second-strand cDNA synthesis, terminal repair, 3’ end A-tailing, ligation, and enrichment, were completed according to the manufacturers’ instructions. Sequencing was performed according to the corresponding requirements with a paired-ending cDNA sequencing program. The sequencing process was controlled using the Illumina HiSeq 2000 platform data collection software program that was used for the real-time data analysis. The raw reads were processed to remove adaptor sequences, low-quality reads and reads containing poly-N sequences with Seqtk^1^. Using the spliced mapping algorithm in TopHat (version: 2.0.9) (Trapnell et al., 2012), the clean reads were mapped to the Ensembl Galgal5 reference genome. The number of fragments per gene was determined using StringTie (version: 1.3.0), and the values were normalized by the trimmed mean of *M*-values (TMM) method (Robinson and Oshlack, 2010). The fragments per kilobase of transcript per million mapped reads (FPKM) value of each gene was calculated using a Perl script (Poelchau et al., 2014).

### Differential Expression and Functional Enrichment Analysis

EdgeR software was used to identify the differentially expressed genes (DEGs) between the control group and test group IV (Haydock et al., 2015). The threshold *P*-value was determined by controlling the false discovery rate (FDR), and the corrected *p*-value was the *q*-value (Kim and van de Wiel, 2008). The fold changes in the gene expression level were calculated based on the FPKM values. The screening conditions for DEGs were a *q*-value ≤0.05 and a fold change ≥2. The functional classifications of the DEGs were analyzed by Gene Ontology (GO) functional enrichment analysis (Young et al., 2010) and KOBAS software (Mao et al., 2005). The GO terms and KEGG pathways with *P*-values <0.05 were considered significantly enriched. The genes in the KEGG pathways related to pigmentation were submitted to STRINGv10.0 for protein-protein interaction (PPI) network analysis (Franceschini et al., 2013), and the PPI network was visualized using Cytoscape (version 3.6.0) (Shannon et al., 2003).

### Quantitative Real-Time PCR (qRT-PCR) Analysis

To verify the accuracy of the RNA-seq results, seven genes were selected for qRT-PCR validation. The gene-specific primers were designed using the NCBI Primer BLAST online program (Supplementary Table S2) and synthesized by Nanjing Kingsray Biotechnology Co., Ltd. The qRT-PCR analysis was performed using a PrimeScript^TM^ RT Reagent Kit and SYBR^®^ Premix Ex Taq II (Tli RNase H Plus; Takara, Dalian, China). The PCRs were conducted with an initial denaturation step at 95°C for 5 min followed by 34 cycles of 95°C for 15 s (denaturation), 60°C for 45 s (annealing), and 72°C for 40 s (extension) and a final extension at 72°C for 10 min. The reactions for each qRT-PCR were performed in triplicate. The relative expression levels of the genes were calculated using the 2^-ΔΔCt^ method, and the levels were normalized to those of two housekeeping genes, *β-actin* and *GAPDH* (Vandesompele et al., 2002).

### Statistical Analysis

All the data were statistically analyzed with SPSS 20.0 software to determine the significance of the differences (*P* < 0.05) between the test groups and the control group. The results are expressed as the mean ± standard deviation (SD).

## Results

### Concentration-Dependent Effect of Dietary Tyrosine on the Breast Muscle Melanin Content

**Figure 1:**
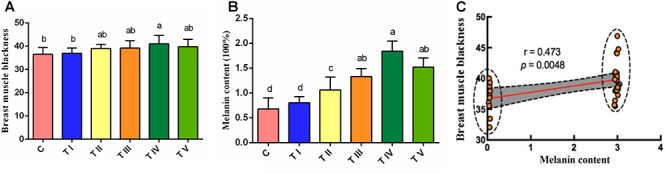
Degree of blackness and melanin content of breast muscle tissue. **(A)** Breast muscle blackness. **(B)** Melanin content of breast muscle tissue. **(C)** Analysis of the correlation between the melanin content and the degree of blackness of breast muscle tissue. In x-axis, the C represents the control group, and the TI-TV represents test groups I-V, respectively. Data are expressed as mean ± SD (*n* = 60). The small letters a, b, etc., on the top of each bar represents statistical significance. Data with different small letters on each bar are statistically significant (*P* < 0.05), while data with the same letters are not statistically significant (*P* > 0.05).

The results indicate that dietary supplementation with different doses of tyrosine significantly improved the breast muscle blackness (Figure 1A) and increased the melanin content in the breast muscle of Xichuan black-bone chickens (Figure 1B). The lowest melanin content in breast muscle was detected in the control group, and a slightly increased content was found in test group I, but no significant differences were found between test group I and the control group (*P* > 0.05). However, the melanin contents of the test groups II-V were significantly higher than those of the control group and test group I (*P* < 0.05), and the maximal value was obtained with 0.8% tyrosine supplementation (Figure 1B). Although the melanin content was also elevated by 1.0% tyrosine supplementation, the content obtained with this level of tyrosine supplementation was lower than that obtained with 0.8% tyrosine supplementation. Thus, the 0.8% dose of dietary tyrosine was identified as the maximal effective dose for promoting the melanin content in breast muscle. In addition, a correlation analysis showed a significant correlation between the breast muscle darkness and the melanin content (*P* < 0.05) (Figure 1C).

### Effect of Tyrosine Exposure on the Proliferation and Tyrosinase Content of Melanocytes

**Figure 2:**
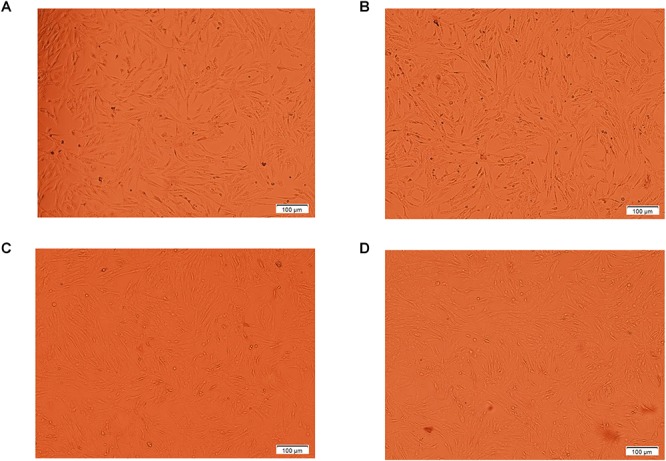
The morphology of melanocytes treated with different concentrations of tyrosine for 72 h were observed by inverted phase contrast microscope. The **A** indicates the control melanocytes. The **B**, **C**, and **D** indicate the melanocytes cultured with 10^-9^, 10^-8^, and 10^-6^ mol/L tyrosine, respectively. The number of cells and cytoplasm in the experimental group increased significantly, especially in the 10^-9^mol/L tyrosine group.

**Figure 3:**
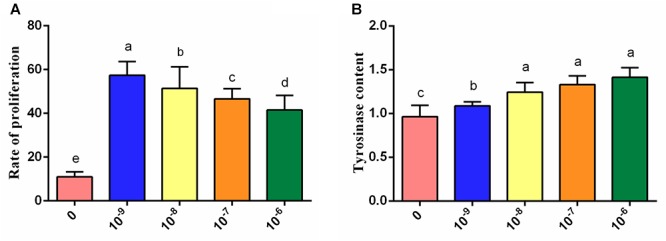
Effects of treatment with different concentrations of tyrosine for 72 h on melanocytes. **(A)** Effects of different concentrations of tyrosine on melanocyte proliferation. **(B)** Effects of different concentrations of tyrosine on the tyrosinase content of melanocytes. Data are expressed as mean ± SD (*n* = 10). The small letters a, b, etc., on the top of each bar represents statistical significance. Data with different small letters on each bar are statistically significant (*P* < 0.05), while data with the same letters are not statistically significant (*P* > 0.05).

We also evaluated the effect of tyrosine in chicken melanocytes. Melanocytes were cultured with different concentrations of tyrosine for 72 h, and the melanocyte morphology was then observed under an inverted microscope. The test groups treated with different concentrations of tyrosine showed significantly higher numbers of cells and evident cytoplasm enlargement compared with the control group. In particular, the most obvious cytoplasmic change was detected in the group treated with 10^-9^ mol/L tyrosine (Figure 2). The analysis of the melanocyte proliferation rates also showed that the group treated with 10^-9^ mol/L tyrosine exhibited the maximal proliferation effect (*P* < 0.05) (Figure 3A). Moreover, the intracellular tyrosinase content was also measured using a microplate reader (Figure 3B), and the results showed that the tyrosinase content in the group treated with 10^-9^ mol/L tyrosine was significantly higher than that in the control group and that the maximal proliferation effect was obtained in the group treated with 10^-6^ mol/L tyrosine (*P* < 0.05). These results indicated that supplementation with an appropriate concentration of tyrosine can significantly increase the proliferation and tyrosinase content of melanocytes of Xichuan black-bone chickens.

### Characteristics of the Transcriptome Profiles of Breast Muscle With the Highest Content of Melanin

**Figure 4:**
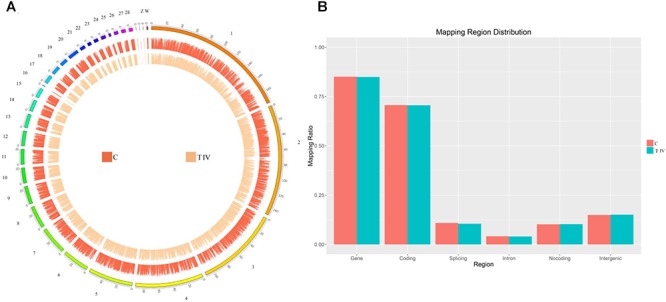
Distribution of the identified mRNAs on each chromosome. **(A)** The outer ring represents the chicken genome and is labeled with the chromosome numbers and positions. The red circle shows the distribution of the mRNAs identified in the control group, and the orange circle shows the distribution of the mRNAs identified in test group IV. **(B)** Mapping region distribution.

The above-described studies demonstrated that tyrosine can promote melanin deposition in the breast muscle of Xichuan black-bone chickens at the cellular level and *in vivo*. To further reveal the molecular regulatory mechanism underlying this biological process, we used the pooled total RNA from breast muscle tissues of the control group and test group IV (0.8% dietary tyrosine level and the highest melanin content) and performed transcriptome sequencing using the Illumina HiSeq 2000 platform. A total of 47,038,307 (95.34%) and 51,161,382 (95.46%) clean reads were obtained for the control group and test group IV, respectively (Table 1). After assembly, 15,074 (94.4%) reads were obtained for the control group, 15,173 (95.02%) reads were found for test group IV, and 14,277 reads were commonly expressed in both groups. These sequencing data have been submitted to the Genome Expression Omnibus (Accession Number GSE128028) of the National Center for Biotechnology Information (NCBI). The mapping rates for the reads that were uniquely mapped to the Galgal5 assembly of the chicken genome were 84.01 and 85.01% for the control group and test group IV, respectively. The comparison of the results aligned to their respective chromosomes showed no significant differences between the samples (Figure 4A). Most of these clean reads mapped to genes and coding regions (Figure 4B), consistent with the results of previous studies (Li H. et al., 2015; Zhang et al., 2017).

**Table 1:** Statistics for the cDNA library sequences.

Sample ID	Raw reads	Clean reads	Mapped reads	Unique mapped reads^a^	Mapping ratio^b^	Number of genes
Control	49,337,216	47,038,307	40,010,067	39,519,161	87.73%	15,074
Treatment IV	53,597,228	51,161,382	44,002,169	43,489,850	88.51%	15,173

^a^*Mapped Unique reads, reads that matched the reference genome in only one position. ^*b*^Mapping ratio, mapped reads/clean reads*.

**Figure 5:**
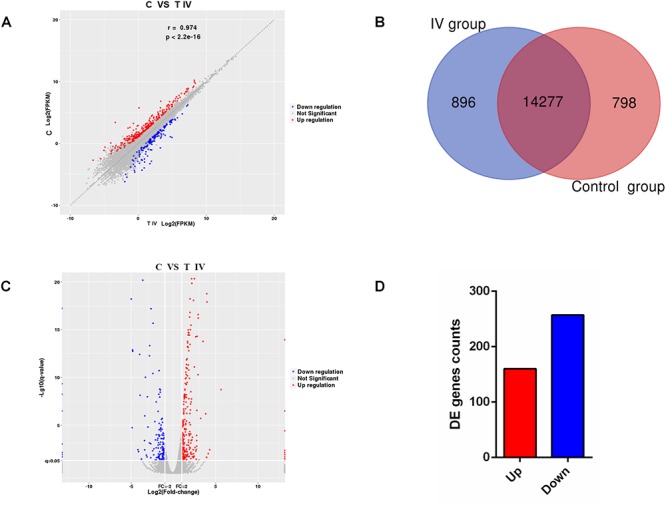
RNA-seq transcriptome profiles distinguishing the two groups. **(A)** Correlations of the gene expression levels among the samples. **(B)** Number of genes identified in breast muscle tissue from chickens in the different groups. **(C)** Volcano plot of differentially expressed mRNAs between the control group and test group IV. **(D)** Total number of differentially expressed mRNAs between the control group and test group IV. Red represents gene upregulation, and blue represents gene downregulation.

**Figure 6:**
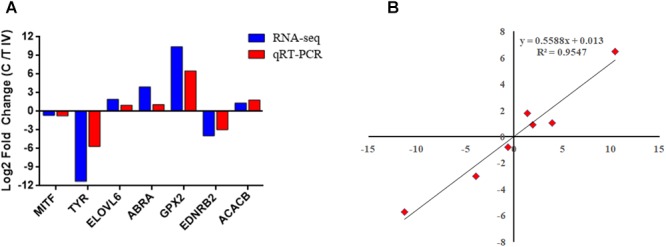
Validation of the RNA-seq data by qRT-PCR. **(A)** Comparison of the fold change between RNA-seq and qRT-PCR. **(B)** Pearson correlation scattered plots based on the results of RNA-seq and qRT-PCR.

The correlation analysis showed that the gene expression data were highly correlated between the two groups, with a correlation coefficient (*R*^2^) of 0.974 (Figure 5A), which indicated the rationality of the sample selection in this study. Among the 15,968 genes identified in the breast muscle of Xichuan black-bone chickens, 14,277 genes were coexpressed in the two groups, 798 genes were specifically expressed in the control group, and 896 genes were specifically expressed in test group IV (Figure 5B). Compared with the control group, 417 DEGs with a fold-change ≥2 and *q*-value ≤0.05 (Figure 5D) were detected in test group IV (Figure 5C and Supplementary Table S3), and these DEGs included 160 upregulated genes and 257 downregulated genes. Moreover, three downregulated (*MITF*, *TYR*, and *EDNRB2*) and four upregulated (*ACACB, ELOVL6*, *ABRA*, and *GPX2*) genes were selected for qRT-PCR verification. The log_2_ (fold change) values of the seven genes obtained from the qRT-PCR analysis were consistent with the RNA-seq results (Figure 6A). An analysis of the correlation between the RNA-seq and qRT-PCR results yielded a correlation coefficient of 0.95, which indicated the reliability of the sequencing results (Figure 6B).

**Figure 7:**
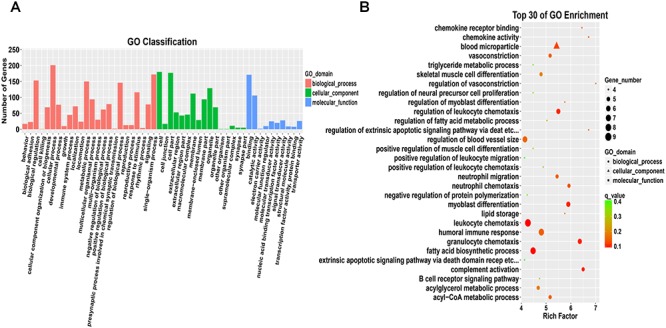
Gene Ontology analysis of differentially expressed mRNAs. **(A)** Comparison of GO annotations of functional genes. The horizontal axis shows the secondary nodes of three GO categories. The vertical axis displays the number of annotated differentially expressed genes. **(B)** Scatter plot of the top 30 enriched GO terms identified from the differentially expressed mRNAs in breast tissue from the control group and test group IV.

**Figure 8:**
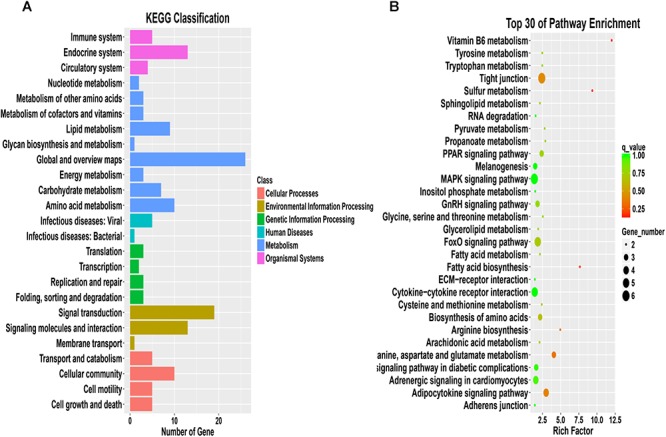
KEGG analysis of differentially expressed mRNAs. **(A)** Comparison of KEGG annotations of functional genes. **(B)** Scatter plot of the top 30 enriched pathways identified from the differentially expressed mRNAs in breast tissue from the control group and test group IV.

A functional enrichment analysis of these 417 DEGs was then performed to identify their physiological functions. The GO enrichment analysis revealed 24 annotations for biological process terms, 16 annotations for cellular component terms and 10 annotations for molecular function terms (Figure 7 and Supplementary Table S4). In addition, these DEGs were enriched in 91 KEGG pathways (Figure 8 and Supplementary Table S5), and these pathways can be divided into six types, namely, cellular processes, environmental information processing, genetic information processing, human diseases, metabolism, and organismal systems.

### Key Genes in Tyrosine-Induced Melanin Deposition

**Figure 9:**
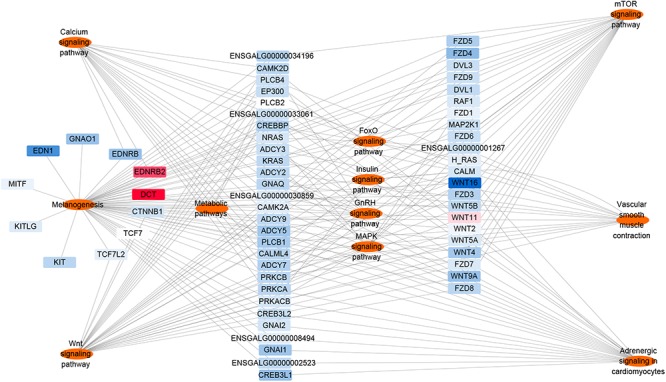
Differentially expressed mRNAs involved in the melanin production pathway. The red nodes represent upregulated genes, and the closer to red indicate that the degree of the up-regulation is higher. The blue nodes represent downregulated genes, and the closer to blue indicate that the degree of the down-regulation is higher.

**Figure 10:**
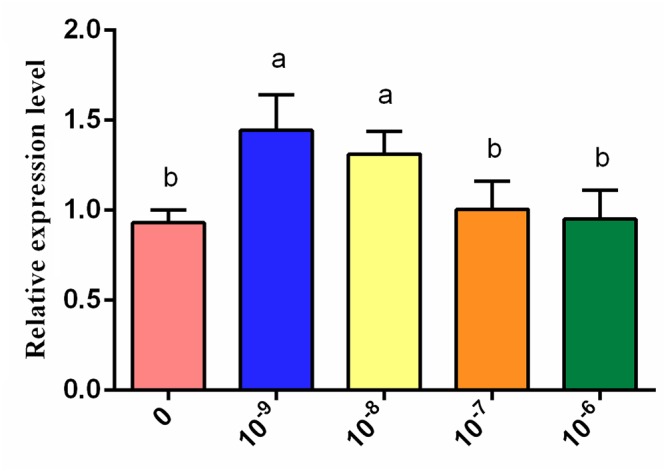
Expression level of the *EDNRB2* gene (determined by qRT-PCR analysis) in melanocytes of Xichuan black-bone chickens treated with different concentrations of tyrosine. Data are expressed as mean ± SD (*n* = 6). The small letters on the top of each bar represents statistical significance. Data with different small letters on each bar are statistically significant (*P* < 0.05), while data with the same letters are not statistically significant (*P* > 0.05).

Based on the results of the KEGG pathway enrichment analysis of the DEGs, some melanin synthesis-related pathways, including melanogenesis, the calcium signaling pathway, the Wnt signaling pathway, the mTOR signaling pathway, vascular smooth muscle contraction and adrenergic signaling in cardiomyocytes, were screened (Figure 9). Some key genes related to melanin synthesis were identified from the above pathways, and these were closely associated with pigmentation (Table 2). Among these genes, *DCT* and *EDNRB2* were identified as genes that participate in many pathways, such as melanogenesis and the calcium signaling pathway. Thus, we focused on the effect of tyrosine on *EDNRB2* gene expression in chicken melanocytes. Under the experimental treatment conditions with different concentrations of tyrosine, the expression of the *EDNRB2* gene was significantly upregulated in a dose-dependent manner in melanocytes of Xichuan black-bone chickens (Figure 10). In particular, the highest *EDNRB2* mRNA expression level was obtained in the group treated with 10^-9^ mol/L tyrosine, and this expression level was significantly different compared with the control group (*P* < 0.05). It is known that the *EDNRB2* gene activates the downstream signaling molecule PKC, upregulates the downstream gene *DCT* and thereby affects melanin deposition.

**Table 2:** Differentially expressed genes related to pigmentation.

Gene name	Full Name	Control group	Test IV group	Log2 (fold change)	*Q*-value
*TRPM1*	transient receptor potential cation channel subfamily M member 1	0.0138	0.2533	-4.19	3.64 E-03
*SOX13*	SRY-box 13	0.6794	2.3681	-1.80	1.21E-03
*PMEL*	premelanosome protein	0.1643	1.3869	-3.07	1.05E-08
*MYOG*	myogenin	1.1535	3.9970	-1.79	2.44 E-03
*MLPH*	melanophilin	0.1330	0.9518	-2.83	7.14 E-04
*MLANA*	melan-A	0.0012	2.0618	-10.69	7.21 E-03
*GREM1*	gremlin 1, DAN family BMP antagonist	1.3070	4.4955	-1.78	1.84 E-02
*GPNMB*	glycoprotein nmb	0.1359	3.8240	-4.81	1.83E-13
*EDNRB2*	endothelin receptor B subtype 2	0.1236	1.9003	-3.94	7.35E-09
*DCT*	dopachrome tautomerase	0.1034	3.2732	-4.98	6.13E-19

## Discussion

Animal phenotypes are influenced by both genetics and the environment. The most obvious difference between black-bone chickens and ordinary chickens is that the skin, muscle, periosteum and other tissues of black-bone chickens contain high levels of melanin (Hirano, 1990; Tian et al., 2007). Therefore, we first investigated the effects of six levels of tyrosine supplementation on melanin deposition in the breast muscles of Xichuan black-bone chickens. The results showed that the effects of dietary tyrosine on the breast muscle melanin content are concentration-dependent. In particular, the basal diet with 0.8% tyrosine significantly improved the blackness degree of breast muscle tissue and yielded the highest melanin content in breast muscle. Previous studies have shown that diets with low levels of tyrosine turn black cat hair a reddish-brown color (Yu et al., 2001), whereas dietary supplementation with sufficient tyrosine can prevent this change (Morris et al., 2002). Our study showed that appropriate tyrosine levels can promote melanin synthesis, which is consistent with the results of previous studies (Tsatmali et al., 2002).

Melanin is a type of high-protein molecule synthesized from melanin bodies in melanocytes (Diffey et al., 1995; Mackintosh, 2001). Black-bone chicken melanin is produced by TYR through a series of biochemical reactions (Sato et al., 2007). Thus, we also evaluated the effects of tyrosine exposure on the proliferation and tyrosinase content of melanocyte. The results indicated that treatment with different concentrations of tyrosine could induce the proliferation of chicken melanocytes cultured *in vitro*. The proliferation of chicken melanocytes increased with increases in the tyrosine concentrations within a certain concentration range, but tyrosine concentrations that exceed a certain range inhibited melanocyte proliferation to a certain degree. We also found that tyrosine dose-dependently affects the tyrosinase content of chicken melanocytes. Specifically, the tyrosinase content of melanocytes was significantly upregulated in the tyrosine-treated groups compared with the control group, and higher levels of tyrosine supplementation increased the intracellular tyrosinase content. Analogously, previous studies have also demonstrated that increases in the tyrosine concentrations can promote melanin synthesis in hamster melanoma cells, and a certain dose-dependent relationship has been found between tyrosine and tyrosinase (Słominski et al., 1988). These studies have indicated that the meat quality, particularly the blackness degree and melanin content, could be improved by the addition of an appropriate amount of tyrosine in Xichuan black-bone chicken farming.

Moreover, we highlighted the potential molecular mechanism underlying tyrosine-induced melanin deposition in the breast muscle of Xichuan black-bone chickens. Some key genes involved in tyrosine-induced melanin deposition, such as premelanosome (*PMEL*), melanophilin (*MLPH*), *EDNRB2*, *DCT*, and other pigment-related genes, were identified based on the transcriptome profiles constructed using the pooled total RNA from the breast muscle tissue of the control group and experimental group treated with 0.8% tyrosine supplementation. Therefore, *PMEL*, which is also known as *gp100*, *ME20*, *PMEL17*, and silver, is a melanoma-specific glycoprotein that plays an important role in the development of melanin bodies by forming a proteolytic fibrous matrix for melanin deposition (Leonhardt et al., 2013). *MLPH*, a small GTP-binding protein (Rab27a) and myosin-Va (Myosin-Va, Myo5a) form ternary complexes that play a key role in the transport of melanosomes (Hume et al., 2007). These complexes aggregate at the dendritic terminals of melanocytes and transfer melanosomes from melanocytes to adjacent keratinocytes. Mutations in the *MLPH* gene might affect the transport of melanosomes. A large number of studies have shown that variations in the *MLPH* gene affect animal hair color and skin color development (Hume et al., 2006; Bed’hom et al., 2012; Lehner et al., 2013; Bauer et al., 2018). Therefore, these genes might play a key role in tyrosine-induced melanin deposition in the breast muscle of Xichuan black-bone chickens.

Among the identified key genes, *EDNRB2* is a paralog of the endothelin receptor B (*EDNRB*) gene and encodes a G protein-coupled receptor (GPCR) with seven transmembrane domains (Lecoin et al., 1998; Pla et al., 2005; Harris et al., 2008; Braasch and Schartl, 2014). Abnormalities in the *EDNRB2* gene cause defects in pigment biosynthesis, which results in the production of pink skin and white fur (Kelsh et al., 2009; Fontanesi et al., 2014; Li L. et al., 2015). *EDNRB2* is also involved in melanocyte differentiation and migration *in vitro* and *in ovo* (Pla et al., 2005). To date, *EDNRB2* has been found to be associated with phenotypes in quail (Miwa et al., 2007), chickens (Kinoshita et al., 2014), frog (Kawasaki-Nishihara et al., 2011) and domestic duck (Li L. et al., 2015). In this study, *EDNRB2* was also found to be upregulated in chicken melanocytes at the transcriptional level. Although the expression levels of *EDNRB2* did not continue to increase with increases in the concentrations of tyrosine, its expression remained significantly higher than that in the group not treated with tyrosine. These studies indicate that *EDNRB2* plays an important role in tyrosine-induced melanin deposition.

**Figure 11:**
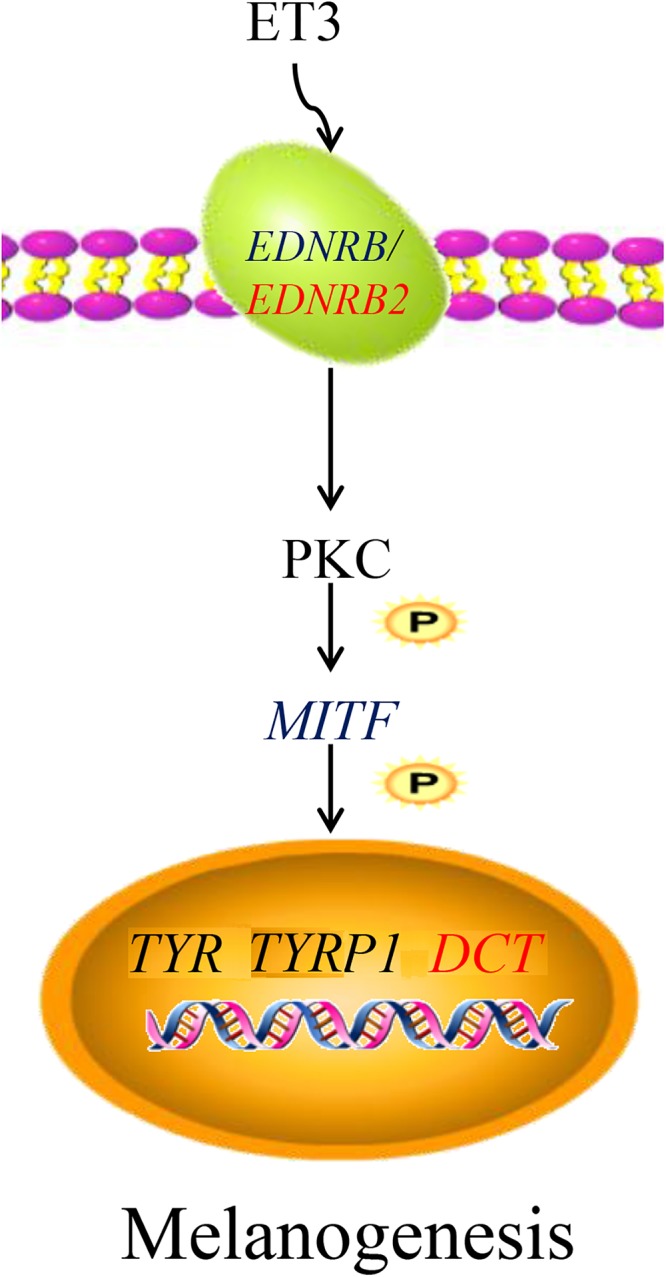
Network of *EDNRB2*-mediated regulation of chicken melanin synthesis. In melanocytes, the *ET3* gene binds to *EDNRB2* gene to activates the downstream signal transduction molecule *PKC*, and the activation of *PKC* activates the expression of many specific genes such as *TYR*, *TYRP1*, and *DCT*. The *DCT* gene can catalyze the transformation of dopamine into 5,6-dihydroxyindole carboxylic acid to accelerate the formation of melanin. *ET3*, endothelin 3; *EDNRB*, endothelin receptor B; *EDNRB2*, endothelin receptor B subtype 2; *PKC*, protein kinase C; *MITF*, melanogenesis associated transcription factor; *TYR*, tyrosinase; *TYRP1*, tyrosinase-related protein 1; *DCT*, dopachrome tautomerase.

Based on the above results and those of a previous study, a network for the *EDNRB2*-mediated regulation of chicken melanin synthesis was constructed (Figure 11), and this network plays an important role in the regulation of melanocyte development (Hou et al., 2004; Saldana-Caboverde and Kos, 2010; Kawasaki-Nishihara et al., 2011). In this proposed network, *ET3*, an isomer of the endothelin receptor, has similar affinities for *EDNRB* and *EDNRB2* and binds to these receptors to activate the downstream signal transduction molecule *PKC*, and the activation of *PKC* in turn triggers a physiological response in the cell (Pla et al., 2005; Kinoshita et al., 2014). This response then activates the expression of many specific genes, such as dopachrome tautomerase (*DCT*) (Guibert et al., 2004; Saternus et al., 2015; Salinas-Santander et al., 2018), tyrosinase (*TYR*) (Guibert et al., 2004; Xu et al., 2013), and tyrosinase-related protein 1 (*TYRP1*) (Alonso et al., 2008; Saternus et al., 2015), in melanocytes. As a dopamine isomerase downstream of tyrosine, *DCT* can catalyze the transformation of dopamine into 5,6-dihydroxyindole carboxylic acid, which can accelerate the formation of melanin and in turn affect melanin deposition in black-bone chicken breast muscle tissue.

## Conclusion

In summary, this study provides the first demonstration that tyrosine can promote melanin deposition in the breast muscle of Xichuan black-bone chickens at the cellular level and *in vivo*. For Xichuan black-bone chickens, the optimal tyrosine dietary supplementation level was found to be 0.8%. We also analyzed the characteristics of the transcriptome profiles of breast muscle tissue from the control group and test group IV (0.8% dietary tyrosine level and the highest melanin content). The *EDNRB2*-mediated regulatory network might play a key role in tyrosine-induced melanin deposition in the breast muscle of Xichuan black-bone chickens. These results provide insights into the molecular regulatory mechanisms for melanin deposition in black-bone chickens and are important for the nutrition regulation and the genetic improvement of the meat quality of Xichuan black-bone chickens.

## Ethics Statement

The whole sample collection and treatment were conducted in strict accordance with the protocol approved by the Institutional Animal Care and Use Committee (IACUC) of Henan Agricultural University, China (11-0099).

## Author Contributions

XK and GL conceived and designed the experiments. YC, JW, and YC contributed to animal husbandry. YZ, YL, and YF performed the experiments. YT and XW analyzed the data. CZ, RJ, and ZL contributed reagents and materials. GS and WL contributed analysis tools. DL wrote the manuscript. All authors reviewed and approved the final manuscript.

## Conflict of Interest Statement

The authors declare that the research was conducted in the absence of any commercial or financial relationships that could be construed as a potential conflict of interest.

## Footnotes

1 https://github.com/lh3/seqtk
