# Supplementary material for: Transcriptome Analysis of the Breast Muscle of Xichuan Black-Bone Chickens Under Tyrosine Supplementation Revealed the Mechanism of Tyrosine-Induced Melanin Deposition
Source: Front Genet. 2019 May 15;10:457. doi: 10.3389/fgene.2019.00457 (PMC6529781; doi:10.3389/fgene.2019.00457)
Supplement: Supplementary file 1 [file Table_1.DOCX]

**Table S1** Composition and nutrient levels of the basal diets (air-dry basis).

| **Ingredients** | **Content (%)** | | **Nutrition level** | **Content(%)** | |
| --- | --- | --- | --- | --- | --- |
|  | **0-6 Week** | **7-12 Week** |  | **0-6 Week** | **7-12Week** |
| Corn | 64.31 | 61.56 | ME（MJ/kg） | 11.75 | 11.55 |
| Soybean meal | 26.72 | 21.2 | CP（%） | 18.6 | 17.88 |
| Wheat bran | 4.46 | 11.9 | Ca（%） | 0.96 | 1.03 |
| Limestone | 0.89 | 0.94 | AP（%） | 0.7 | 0.76 |
| NaCl | 0.35 | 0.34 | Met (%） | 0.28 | 0.82 |
| Ca HPO4 | 1.02 | 2.02 | Lys (%) | 0.9 | 0.78 |
| Fish mea | 0.99 | 0.98 |  |  |  |
| Premix | 1 | 1 |  |  |  |
| Total | 100 | 100 |  |  |  |

Note: The premix contains vitamins, trace elements, lysine, methionine and threonine. Metabolic energy, crude protein, calcium and available phosphorus were calculated, and amino acid content was measured.
