# Supplementary material for: Transcriptome Analysis of the Breast Muscle of Xichuan Black-Bone Chickens Under Tyrosine Supplementation Revealed the Mechanism of Tyrosine-Induced Melanin Deposition
Source: Front Genet. 2019 May 15;10:457. doi: 10.3389/fgene.2019.00457 (PMC6529781; doi:10.3389/fgene.2019.00457)
Supplement: Supplementary file 2 [file Table_2.DOCX]

**Table S2** Information on the qPCR primers used in this study.

| **Gene** | **Transcript ID** | **Sequence (5’ to 3’)** | **Product size（bp）** | |
| --- | --- | --- | --- | --- |
| *MITF* | NM_205029.1 | F:AGCTCACAGAGTCAGAAGCG  R:TATTCCAGCGCATATCCGGG | 159 |  |
| *TYR* | NM_204160.1 | F:TTGACAGCATTTTTGAGCGGTG  R:CTGATGGGCTTGCTTGAGGT | 242 |  |
| *EDNRB2* | NM_204120.1 | F:TGAGCAAGAGAAATGGCATGA  R:CCTCCCGACGCCGTTT | 60 |  |
| *ACACB* | XM_015275512.1 | F:CAACTTGGGGACAAATTGGG  R:CACCACCCATCATCTCCGA | 101 |  |
| *ELOVL6* | NM_001031539.1 | F:CATAGCTCAGGGTCATC  R:TTCTGGAGCAGCAG | 225 |  |
| *ABRA* | XM_428503.5 | F:GACAATGGCCGAGAGGTGAC  R:AACGGATTCAGCTTCTGCGT | 173 |  |
| *GPX2* | NM_001277854.1 | F:CGCCAAGTCCTTCTACGACC  R:GGTGTAATCCCTCACCGTGG | 133 |  |
| *β-actin* | NM_205518.1 | F: GAGAGAAGATGACACAGATC  R: GTCCATCACAATACCAGTGG | 116 |  |
| *GADPH* | NM_204305.1 | F: TGATGGTCCACATGGCATCC  R: GGGAACAGAACTGGCCTCTC | 141 |  |
